# Guess Who’s Back: Persistence and Circulation of *Salmonella* Infantis on Broiler Farms with a History of Contamination

**DOI:** 10.3390/foods15020339

**Published:** 2026-01-17

**Authors:** Lisa De Witte, Koen De Reu, Maxim Van der Eycken, Joke Van Raemdonck, Nadine Botteldoorn, Filip Van Immerseel, Geertrui Rasschaert

**Affiliations:** 1 Technology and Food Unit, Flanders Research Institute for Agriculture, Fisheries and Food (ILVO), 9090 Merelbeke-Melle, Belgium; lisa.dewitte@ilvo.vlaanderen.be (L.D.W.); koen.dereu@ilvo.vlaanderen.be (K.D.R.);; 2 Dierengezondheidszorg Vlaanderen (DGZ), 8820 Torhout, Belgiumnadine.botteldoorn@dgz.be (N.B.); 3 Faculty of Veterinary Medicine, Ghent University (UGent), 9820 Merelbeke-Melle, Belgium; filip.vanimmerseel@ugent.be

**Keywords:** *Salmonella*, Infantis, broiler farm, cleaning and disinfection, biosecurity

## Abstract

For several years, Infantis was the most common *Salmonella* serovar circulating in the Belgian broiler sector and persisting on broiler farms. To gain insight into its prevalence and circulation on broiler farms in Belgium, five farms (14 flocks) with a *S.* Infantis contamination history were monitored during two consecutive production rounds. In total, ten sampling events were conducted using moist sponge sticks after cleaning and disinfection, during the delivery of the one-day-old chicks and during production until slaughter or until positive for *S.* Infantis. *Salmonella* presence on samples was determined based on the ISO 6579:2017 standard, and the isolated strains were typed using PFGE. The results showed that current cleaning and disinfection practices were unable to completely remove *S.* Infantis from the farms. Cleaning equipment (3 out of 9 sample times) and the floor (5 out of 10 sample times) were particularly contaminated. Furthermore, external environmental samples were also frequently contaminated (e.g., mortality containers, concrete driveway). During production, 12 of the 28 sampled flocks were colonized with *S.* Infantis after one week, indicating that *S.* Infantis quickly spreads throughout the broiler house, which raises the hypothesis that feeding and/or drinking water systems play a critical role in the circulation of the bacteria. This study gives insights into the circulation and difficulty of controlling *S.* Infantis in persistently contaminated broiler farms, highlighting the importance of thorough cleaning and disinfection and biosecurity.

## 1. Introduction

In the European Union, human infections with *Salmonella* have been increasing in the period 2020–2024 [1]. In case of infection, *Salmonella* causes salmonellosis, which is the second most commonly reported zoonosis in humans in EU [1]. It manifests as a self-limiting gastroenteritis in most people; however, several groups of people (young, old, pregnant, and immunosuppressed people) are at risk for more severe infections that could require antibiotic treatment, which can be compromised by antibiotic resistance [2]. In 2024, *Salmonella* Infantis (SI) remained a critical human health threat because of its prevalence in broiler flocks and broiler meat in combination with high levels of antibiotic resistance [1]. With 1192 human cases in EU in 2024, Infantis is currently the fourth most frequent *Salmonella* serovar isolated from humans [1]. In broiler flocks, on the other hand, Infantis is the most common *Salmonella* serovar, with more than 57% of the serotyped European isolates [1]. Also, in Belgium, Infantis is one of the most common *Salmonella* serovars. In the years prior to the start of this research, 2017–2021, Infantis was the most frequently isolated serovar of *Salmonella* in Belgian broiler flocks, with a prevalence fluctuating between 30 and 38% [3]. With 29% of the *Salmonella*-colonized broiler flocks belonging to serovar Infantis in 2023, it is now the second most common serovar after Paratyphi B var. Java [3]. Despite a national control program (NCP) in place, between 70 and 133 flocks have been found contaminated with SI each year since 2016, without evidence of a decreasing trend [3]. This situation allows for the persistence of SI in the environment of broiler farms, facilitating transmission to future flocks [4]. To reduce the threat to human health, the persistent problem of SI at farm level has to be dealt with, because the transmission and cross-contamination of the bacterium causes its circulation and spread through the poultry food chain [5,6].

Another factor that explains the remarkable ability of SI to persist and spread within the broiler sector is the presence of a megaplasmid, pESI [7]. Previous in vitro research demonstrated the pESI enhances the biofilm formation of SI, its adhesion, and the invasion of avian and mammalian host cells, and increases its tolerance to stress factors [8]. Furthermore, the pESI carries several antibiotic resistance genes, explaining the high levels of antibiotic resistance in SI [8]. In a more recent study, the increased persistence and virulence of a pESI-carrying SI strain compared to a different, pESI-free SI strain were confirmed in an in vivo set-up [9].

*Salmonella* Infantis can be introduced and spread in broiler flocks both vertically (breeder flocks) and horizontally (broilers in the flock and the environment). Vertical transmission has already been discussed in the literature as a risk factor for SI contamination [7,10,11]. Furthermore, SI was isolated from 10 breeding flocks across Europe in 2024, indicating some potential for the vertical transmission of SI [1]. Horizontal transmission, on the other hand, can occur through several routes, such as contaminated feed, water, wild birds, rodents, contaminated equipment and utensils [12,13]. Studies using surveillance data, based on fecal samples taken three weeks before slaughter, show that important risks for SI contamination and persistence in broiler flocks are overall farm management, thinning, and the combination of having temporary workers and contact with foreign or external poultry or persons [5,14].

However, detailed longitudinal data on the specific contamination points and circulation dynamics of SI in persistently infected broiler farms in Belgium are lacking. To gain further insight into contamination risks and elucidate the circulation of SI at broiler farms, this study intensively sampled broiler farms with a contamination history, which we defined as at least two consecutive flocks positive for SI, in Belgium. Furthermore, the occurrence of the megaplasmid in the SI isolates was investigated. It is hypothesized that biosecurity and cleaning and disinfection (C & D) play an important factor in the persistence of SI; furthermore most of the isolates will contain the megaplasmid.

## 2. Materials and Methods

### 2.1. Sampling

Between March 2022 and December 2024, Belgian broiler farms spread over the Flemish region with a SI contamination history were intensively sampled during two consecutive production rounds. The farmers took part in this study on a voluntary basis. During the two rounds, environmental swab samples were collected using 3M sponge sticks (St. Paul, MN, USA) at different timepoints; (1) after C & D of the farms, (2) during delivery of the one-day-old broiler chickens, and (3) every week during rearing until slaughter or until the broiler houses became contaminated with SI. The sponge sticks were premoistened with neutralizing buffer to sample after C & D, and buffered peptone water (BPW, Oxoid, Basingstoke, UK) for sampling the other timepoints. Also, during partial depopulation, samples were collected and the results were discussed by De Witte et al. [15]. The same group of four sampling staff was used for all samplings across all farms. After C & D, an average of 126 samples per farm were collected from all the surfaces and equipment in the broiler houses, the anterooms, and the direct external environment of the broiler houses (Table A1). During delivery of the one-day-old chickens, on average, 24 samples were collected from the crates, inlays and trucks (Table A2). Further, during rearing, on average, 22 samples per broiler house were collected from the surfaces and materials in the broiler houses and anterooms (Table A3), including 2 pairs of overshoes to determine the *Salmonella* status of the broiler chickens, as mentioned in commission regulation (EU) no. 200/2012. All samples were transported in an insulated cooling box and analyzed directly in the lab or kept cooled overnight for analysis the next day.

Information concerning the farms, common practices, and C & D methods was collected using a questionnaire filled out by the farmers (Table 1). All the farms had pre-broiler-house hygiene areas (anterooms), all-in all-out principles, closed mortality containers, and the broiler houses were visited by the farmers daily. The C & D protocols varied between the different farms based on the information given in the questionnaire. Cleaning was performed using an alkaline cleaning product, and the disinfection products used by the farmers consisted of similar active components. However, farm A used an oxidizing disinfection product (peracetic acid and hydrogen peroxide) alternating with disinfection products consisting of QAC, aldehydes and formaldehyde, or alcohols. Farm B also alternated between an oxidizing product and a product combining QACs and aldehydes, but farm C only used a disinfection product consisting of QACs, aldehydes, and alcohols. Farm D used formaldehyde and ammonia, while farm E only used an oxidizing disinfectant.

### 2.2. Salmonella Control on the Farms

Three remedial (*Salmonella* control) methods were tested. At farm C, the internal feeding system was renewed because it was hypothesized that the SI contamination started after a vermin problem in the feeding silo. Critical areas, namely the feeding system, drinking water system, and ventilation system, were intensely sampled at farm D because it was hypothesized that contamination in these systems could explain the early colonization of the broiler chicken. At farm E, an extra sampling moment after cleaning but before disinfection was implemented to evaluate the cleaning step. As before, sponge sticks premoistened with neutralizing buffer or BPW were used to sample the surfaces and equipment. At farm C, the feeding silo was sampled before and after C & D (*n* = 25). The old internal feeding system/line was also sampled after disassembly (*n* = 24). At farm D, the entire feeding system (*n* = 27), drinking water system (*n* = 9), and ventilation system (*n* = 11) were sampled after C & D. At farm E, the ‘after C & D sampling’ protocol (Table A1) was used for the extra sampling event after the cleaning step. The remediations were evaluated by sampling five pairs of overshoe samples every third week of the three consecutive production rounds.

### 2.3. Microbiological Analysis

The sponge sticks swab samples and overshoes were enriched with, respectively, 90 mL and 225 mL BPW, and a bag mixer homogenized the samples. They were further analyzed based on the ISO 6579:2017 standard [16]; briefly, the BPW-enriched samples were incubated for 18 h ± 2 h at 37 ± 1 °C. Afterwards, 100 µL of each sample was distributed with 3 drops on a modified semisolid Rappaport Vassiliadis (MSRV, Oxoid, Basingstoke, UK) agar plate and incubated for 24 and 48 h ± 3 h at 41.5 °C ± 1 °C. Suspected zones on the MSRV plate were sampled by dipping a 10 µL loop in the edge of the opaque halo growth, and were plated on xylose lysine deoxycholate (XLD, Oxoid, Basingstoke, UK) agar. The XLD plates were incubated for 24 h ± 3 h at 37 ± 1 °C. Subsequently, the plates were inspected for characteristic *Salmonella* growth that typically has a black center with a reddish, transparent background on XLD. The suspected colonies were further purified and stored in brain–heart infusion broth (Oxoid, Basingstoke, UK) with 15% glycerol at −20 °C.

### 2.4. Molecular Analysis

#### 2.4.1. Identification by PCR

*Salmonella* suspected isolates were lysed in 50 µL 0.25% NaOH (Merck kGaA, Darmstadt, Germany) and 50 µL 0.25% SDS (Sigma-Aldrich, Saint Louis, MO, USA) at 95 °C for 10 min. These lysates were used in a SI-specific PCR to confirm the serovar Infantis [17]. Isolates that tested negative for the serovar Infantis were then subjected to a *Salmonella* genus PCR to confirm the *Salmonella* genus [18].

#### 2.4.2. Fingerprinting by PFGE

As performed in De Witte et al. [19], isolates confirmed as *Salmonella* were further typed by pulsed-field gel electrophoresis (PFGE) according to the international Pulsenet protocol [20]. A digital picture was taken using the Bio-Rad GelDoc Go Imaging System. The obtained PFGE results were processed in BioNumerics 8.1.1 (Applied Maths NV, Sint-Martens-Latem, Belgium). An unweighted-pair group method using an arithmetic averages (UPGMA) dendrogram was constructed using the default settings of BioNumerics 8.1.1.

#### 2.4.3. Identification of the Megaplasmid

In order to determine the presence of the megaplasmid (pESI), three PCRs were performed using the primer pairs hyp_pESI, Fim and K88, genes that are located on the megaplasmid [8,21]. Furthermore, a PFGE assay was performed to complement the results of the PCR analysis. The same plugs, prepared with the previous PFGE, were used, and as a restriction, the enzyme S1 nuclease (GE Healthcare, Diegem, Belgium) was used, according to the PulseNet protocol [22]. *Salmonella* serovar Braenderup H981, digested with XbaI, was used as a size marker [23,24]. The expected size of the pESI megaplasmid was ~280–310 kb.

### 2.5. Statistical Analysis

Ninety-five percent confidence intervals for proportions were calculated using the Wilson score method without continuity correction and visualized as error bars. Differences between groups were assessed using Fisher’s exact tests. All analyses were performed using R (version 2025.09.1).

## 3. Results

**Figure 1:**
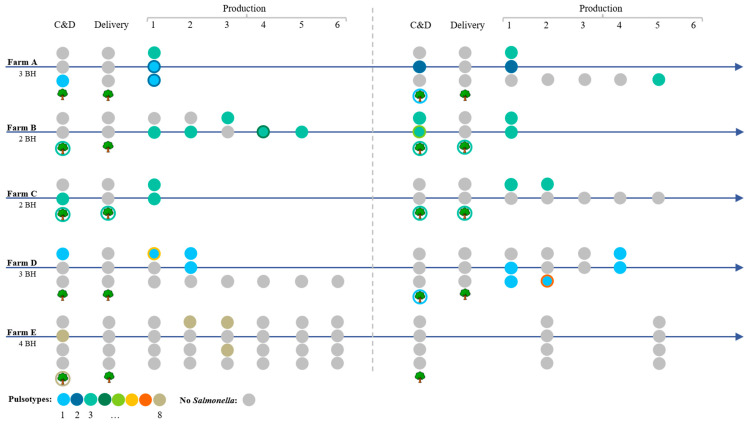
Overview of the results of the *Salmonella* Infantis (SI) circulation at contaminated farms. Dots indicate a sampling event, and a colored dot indicates that SI was found in the broiler house (BH) or anteroom samples; the color indicates which pulsotype (1 until 8). A tree with a colored border indicates that external environmental samples were found contaminated. When more than one pulsotype was present, the filling and outline of a dot have different colors.

A schematic overview of the results is given in Figure 1 and the data is also numerically provided in Table A4.

### 3.1. Farm A

After C & D on farm A, only 1 of 167 samples (0.6%) was SI-positive, originating from residual dirt in broiler house 3 (pulsotype 1). No SI was detected on the delivery material (n = 33) during delivery of the one-day-old chickens, but within the first week, 44 of 75 samples from all three broiler houses were already contaminated with SI, including both overshoe pairs from each broiler house. In broiler house 1, all isolates belonged to pulsotype 3, whereas in broiler houses 2 and 3, pulsotypes 1 and 2 were found. Since all broiler houses were contaminated after one week, no further samples were taken during production. After C & D, before the second production round, 2 of 151 samples (1.3%) were SI-positive, including the drain in broiler house 2 (pulsotype 2) and residual cleaning water in broiler house 3 (pulsotype 1). Again, no SI was detected during delivery (n = 21). After the first week of rearing, 21 samples (including the 4 overshoe samples) from houses 1 and 2 were SI-positive, this concerned pulsotype 3 in broiler house 1 and pulsotype 2 in broiler house 2. Broiler house 3 remained SI-free until the fifth week of rearing, when both overshoe samples and drinking water nipples became contaminated (pulsotype 3). All the isolates obtained from farm A (n = 71) contained the megaplasmid.

### 3.2. Farm B

After C & D at farm B, no SI was isolated from the broiler houses. Only one sample from the inside of the mortality containers was SI-positive, belonging to pulsotype 3 (0.8% of 120 samples). During delivery of the one-day-old chickens, no SI was detected on the inlays or materials (n = 20). In the first week, one sample was SI-positive (pulsotype 3)—the outside surface of the pipes on the wall in broiler house 2. This was again the only positive sample in the second week in the same house, while the other house remained negative. Remarkably, in the third week, house 2 was SI-free, while 14 samples, including both pairs of overshoes, from house 1 were contaminated with SI (pulsotype 3). In the following weeks, no further samples of house 1 were taken, and only house 2 was further sampled weekly. In week 4, both pairs of overshoes and 4 environmental samples were positive for SI pulsotype 3, and one environmental sample was positive for SI pulsotype 4. In the 5th week, 15 samples were positive for SI pulsotype 3. After depopulation, C & D was performed by an external company. Nevertheless, the farm was visually unclean afterwards and the farmer was not satisfied with the cleaning results, resulting in nine samples in the broiler houses and anterooms (seams and gates of both broiler houses, a broom, cracks in the wall and loose dirt in broiler house 2, the computer panel and the door of the anteroom) and one external environmental sample (forks of the forklift) being SI-positive. So, in total, 10 of 128 (7.8%) samples were SI-positive after C & D. With the exception of one isolate (pulsotype 5), all isolates belonged to pulsotype 3. During delivery of the one-day-old chickens, 1 of the 19 swab samples was SI-contaminated, i.e., the wheels of the truck. Important, the wheels were sampled after the truck entered the farm environment. After the 1st week of rearing, 15 samples (including both overshoe samples) in both houses were SI-positive (pulsotype 3). Because the broilers in all houses were already colonized, no further samples were taken in the following weeks. All the isolates sampled at farm B (n = 65) contained the megaplasmid.

### 3.3. Farm C

At farm C, the C & D was performed by the farmer. Although the broiler houses were visually clean after C & D, SI was still found in 14 of the 99 (14.1%) samples. These concerned cleaning materials such as a broom, squeegee, and pressure washer in the anteroom of house 1, and the floor (cracks and seams), walls (cracks), feeding lines, a fan, and ventilation valves in house 2. From the external environment, the inside of the mortality containers were still contaminated. During delivery, SI was isolated from the wheels of the truck, the wheels of the crates, and a fecal dropping in the external environment of the broiler houses (n = 22). As mentioned before, the wheels were sampled after entry to the farm. After the first week, 19 samples of both houses were SI-positive, including all overshoe samples. Since both flocks were already colonized at the age of one week, no further samples were taken. For the second round, samples were taken after C & D and then, only one external sample, the tractor shovel, was found contaminated (1.2% on a total of 86 samples). During delivery, the wheels of the truck were again contaminated (n = 19). In the first week, one pair of overshoes in house 1 was SI-positive. In the second week, 12 samples in house 1 and both overshoe samples were SI-positive. House 2 remained SI-free for the entire rearing period. All isolates from farm C belonged to pulsotype 3 and contained the megaplasmid (n = 53).

### 3.4. Farm D

After C & D in the first round, 2 of 158 (1.3%) samples were SI-positive (pulsotype 1) and were collected from dirt on the floor in broiler house 1 and a squeegee in the anteroom. During delivery, no SI was found on the materials (n = 33). After one week, three samples (boots, buckets, and floor) in the shared anteroom of houses 1 and 2 were positive for SI pulsotypes 1 and 6. Also, in house 1, one pair of overshoes was SI-positive (pulsotype 1). After two weeks, the boots and buckets were again SI-positive, but at that stage, also several materials and all overshoe pairs in houses 1 and 2 were positive for SI pulsotype 1. Broiler house 3 remained SI-free for the remainder of the rearing period. For the second round, SI (pulsotype 1) was again found after C & D, on the floor in the anteroom of house 3 and the external (un)loading area (2 out of 148 samples; 1.4%). No SI was found during delivery (n = 29). After the first week, one pair of overshoes in house 2 and one in house 3 was contaminated with SI (pulsotype 1). In the second week, SI spread in house 3, and both pairs of overshoes, the drinking water nipples, and buckets were contaminated. From the buckets, pulsotype 7 was isolated; the other isolates were of pulsotype 1. In week three, SI pulsotype 1 was isolated from the floor in the shared anteroom of houses 1 and 2, and in week four, the overshoes and 14 other samples of houses 1 and 2 tested SI-positive (pulsotype 1). All the isolates from farm D (n = 43) contained the megaplasmid.

### 3.5. Farm E

To gain insight into critical contamination areas and optimize the cleaning and disinfection, samples were taken after cleaning but before disinfection; 8 of 103 samples (7.8%) were SI-positive: the floor in anteroom 2, boots in anteroom 1, the drains in houses 1 and 2, the gate in house 2, and a pressure washer. Seven days later, after C & D, the floor in house 2 and the (un)loading area in front of house 2 were the only SI-positive samples (1.9%). During delivery, no SI was detected (n = 18). After the first week of rearing, all broiler houses remained SI-free. After the second week, the feeding pipes in house 1 became contaminated. In the third week, SI was found in three samples—one in the anteroom, and one in two of the four different broiler houses. Afterwards, no SI was detected in the four houses for the remainder of the rearing period. After a thorough C & D for the second round, no SI was detected in the 93 samples taken after C & D. Also during rearing, no SI was isolated. All isolated SI in the first round of farm E were of pulsotype 8. Importantly, none of the isolates of farm E (n = 14) contained the megaplasmid.

### 3.6. Contaminated Samples

**Figure 2:**
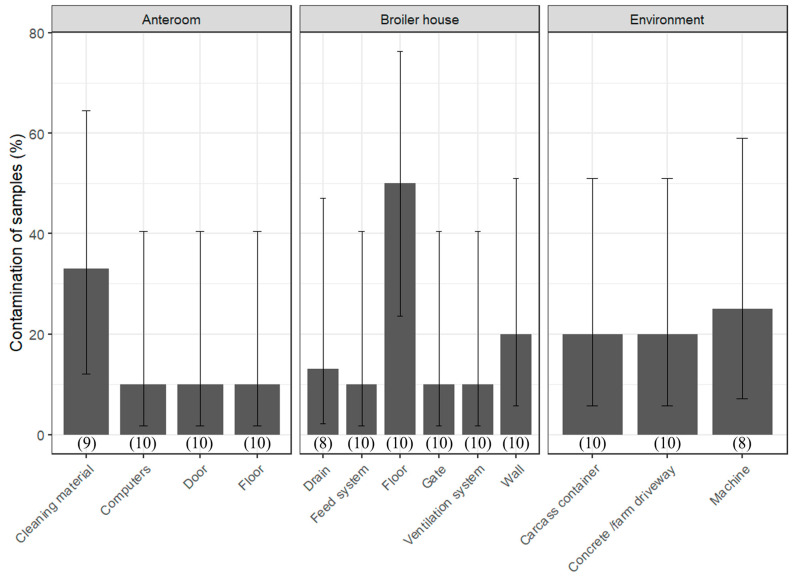
Critical areas and their contamination frequency after cleaning and disinfection in all the farms (A–E). Error bars represent the 95% confidence interval and indicate the uncertainty of the contamination frequency. The number in between brackets indicates the number of sampling events across all farms and rounds in which this sample was taken; the maximal number is 10, indicating that a sample was taken during both production rounds on all five farms. This graphic indicates the critical, frequently SI-contaminated areas; samples that were never contaminated are not shown. This explains the high proportion of contamination compared to the overall contamination rate of 3%.

After C & D on all the farms, SI was still isolated from some critical areas (Figure 2, Table A4). On average, 125 samples were taken and 3.0% of the samples were positive. A standard deviation of 4.2% shows high variability between the farms. Most frequently contaminated were the floors in the broiler houses—specifically, this concerns cracks, seams, and loose dirt. Furthermore, the cleaning materials, such as brooms, squeegees, and high-pressure washers, were also frequently contaminated. Also, the external environmental samples, such as the machines, concrete/farm driveway, mortality containers, and the walls (cracks and seams) in the broiler houses, were sometimes contaminated. Less often contaminated were the ventilation system (openings and ventilator), closing rubber of the gate, feeding system, and drains in the broiler house. Doors, floors, and computers in the anteroom were also not often found to be contaminated. A simple statistical analysis showed no significant differences in the contamination frequency of the sampled materials (*p*-value = 0.6582), which is probably due to the small sample size.

**Figure 3:**
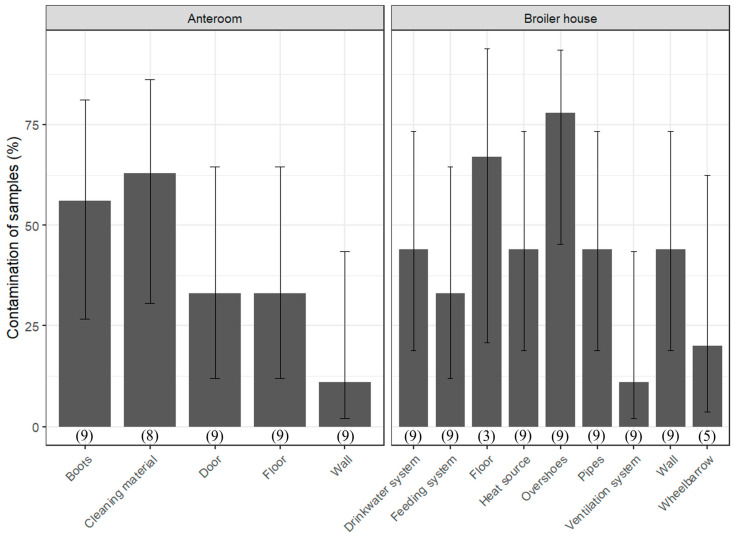
Critical areas and their contamination frequency after the first week of production on all the farms (A–E). Error bars represent the 95% confidence interval and indicate the uncertainty of contamination frequency. The number in between brackets indicates the number of sampling events across all farms and rounds in which the sample was taken; the maximal number is 10, indicating that a sample was taken during both production rounds on all five farms.

During the first week of production, on average, 60 samples were taken, of which 19.2% was contaminated with SI. A standard deviation of 20.3% again shows high variability between the farms. Most frequently contaminated (>50%) were the overshoes and floor in the broiler houses, and cleaning materials (buckets, broom, squeegee) and boots in the anterooms (Figure 3, Table A4). Also frequently contaminated (>30%) were the wall, pipes, the heating system, drinking water system, and feeding system in the broiler houses, and the doors and floor in the anteroom. Less frequently contaminated (<30%) were the wheelbarrow and ventilation system in the broiler houses, and the wall in the anteroom. Again, a simple statistical analysis showed no significant differences in the contamination frequency of the sampled materials (*p*-value = 0.214), which is probably due to the small sample size.

### 3.7. Remediation

At farm C, no SI was detected in the feeding silo before and after C & D, and in the old internal feeding system. The broilers remained SI-free in the consecutive production rounds. At farm D, all samples of the presumptive critical areas were SI-free. However, the broiler chickens were colonized with SI in the next rounds. At farm E, 8 of 103 samples (7.8%) were SI-positive after cleaning and prior disinfection; boots, drains in the broiler houses, gates and gate sealing in the broiler house, and pipes on the wall and floor in the anteroom. No SI was detected using overshoes in the consecutive rounds.

## 4. Discussion

Infantis is the most common serovar colonizing broilers in the European broiler sector [3,25], posing a risk for human salmonellosis. In particular, the presence of the megaplasmid pESI that lends antibiotic resistance genes to the *Salmonella* serovar, makes SI a public health concern [4,26]. In this study, the prevalence and circulation of SI on broiler farms with a history of SI contamination was analyzed.

With one exception, SI was detected after C & D on all the farms. Overall, the highest proportion of positive sample events (21%) originated from the external environment, consistent with the findings from in-depth research of *Salmonella* contamination on broiler farms in Great Britain and other studies [7,27,28]. Newton et al. [28] reported few *Salmonella*-positive samples in anterooms, with floors being the most frequently contaminated surfaces. Similarly to our observations, Newton et al. [28] also identified the contamination of cleaning equipment on several farms. Furthermore, SI persisted on surfaces within the broiler houses during six of the nine sampling events, predominantly on floors (five out of ten samplings), in line with the study of Newton et al. [28], who likewise found floors to be the primary site of contamination inside broiler houses. Possible explanations for these observations are the difficulty of cleaning porous and cracked surfaces and the ability of SI strains to form biofilms [4,8]. Overall, the presence of SI after C & D indicates that current C & D practices, performed by farmers as well as external cleaning firms, are insufficient at removing persistent SI from the farms. The used products do not seem to be the reason for this failure, but rather the lack of a thorough-enough cleaning, since especially cracks, seams, loose dirt, and cleaning equipment were contaminated. In particular, the contamination of cleaning equipment is striking, as it is a critical and yet easy way to avoid the risk of re-contamination in broiler houses. However, the small sample size requires caution when generalizing these observations, especially since several studies have demonstrated that external cleaning services reduce the risk of SI persistence [5,14]. Also, Cawthraw et al. [7] demonstrated that, with adequate C & D, SI can be effectively removed from the broiler houses. However, they also showed that SI can persist in the external environment and in the anteroom.

During production, notably, the overshoe samples in the broiler houses were found positive for SI, an observation shared by Perilli et al. [29]. When a flock became colonized with SI, the number of positive samples quickly increased. Cargnel et al. [12] also described a significant increase in positive samples after SI colonization during broiler and layer production. This rapid spread of SI through the broiler houses can be facilitated by contamination of the feed and drinking water system. Indeed, the drinking water system and feed system in our sampled farms were often found contaminated when the broiler chickens were colonized with SI. As such, these systems can act as a vehicle for the SI contamination already present in the broiler house. In the anteroom, cleaning materials, especially buckets and boots, were frequently found positive with SI during production. Research described that, despite *Salmonella* contamination in the anteroom, the presence of an anteroom still reduces the risk for *Salmonella* contamination of the flocks [28]. Farms with an absence of anterooms or shared anterooms have a higher chance of being *Salmonella*-positive [28]. However, another study showed that the presence of an anteroom at the farm reduces the risk of SI contamination when the flocks are SI-free, but increases the risk of SI colonization when the previous flock was colonized [5]. An important factor seems to be the level of biosecurity that is maintained in the anteroom [27,28,30]. Mughini et al. [14] identified biosecurity as a most important factor for control of SI contamination on the farms, since most of the risk factors seemed to be related to biosecurity compared to other farm characteristics. However, biosecurity entails more than a hygiene barrier in the anteroom, as the movement of personnel and vehicles can also introduce or distribute SI on a farm [28]. As SI can persist in the direct environment, broiler houses can be re-contaminated through clothing, footwear, dust, or aerosols [7].

During delivery, no *Salmonella* was observed on the materials that came with the one-day-old chickens. No contamination was found on the inlays that were sampled to represent the *Salmonella* status of the chickens. Vertical transmission did not appear to be a major contributor in this study, but cannot be excluded, because this is based on a very limited sample size, since farms B, C, and D receive their chicks from the same hatchery. Several studies discussing the impact of vertical transmission of *Salmonella* have conflicting conclusions [31]; a meta-analysis study with an emphasis on US factors, found that the hatchery is the most significant contributor to *Salmonella* contamination. Other studies, focusing on the situation in the UK, found low-level *Salmonella* contamination in hatcheries [32], or found no SI at all [7,27,28]. Different policies in the sampled countries (US vs. Europe) might be the cause for this discrepancy, and the low *Salmonella* prevalence in the UK (in 2020) is probably the results of European Commission Regulation No 200/2010, which includes Infantis as one of the target serovars in the control programs in breeding flocks.

Overall, 94% of the SI isolates carried the megaplasmid pESI, which is similar to the prevalence of pESI in SI isolates from broiler farms in the Netherlands [14]. It is notable that the SI isolates of farm E, the only SI strain that did not contain the megaplasmid, was less prevalent and did not persist during a second production round. However, to combat a simultaneous *Enterococcus* infection at the farm, the antibiotic amoxicillin was applied during the third week of the first production round. The absence of the megaplasmid rendered the SI strain susceptible to the antibiotic, since the resistance gene for this antibiotic was found on the megaplasmid in other strains. Therefore, it is possible that the natural persistence capacity of this strains was masked by the antibiotic treatment. As described in other papers, these results indicate that the spread of SI throughout the broiler sector is parallel to the introduction and spread of the megaplasmid in this serovar [7,33].

## 5. Conclusions

From the results, it can be concluded that SI mainly persists in the external environment surrounding the broiler houses, and current C & D practices can fail to remove SI from the farm. This underscores the importance of strict biosecurity to avoid dragging SI from the environment into the houses. Furthermore, it should not be forgotten that the disinfection is only effective when a proper and thorough clean was performed. Evaluating and improving the cleaning step could optimize the disinfection. Furthermore, horizontal transmission of SI appears to a more important route of re-infection and within-farm spread in these persistently contaminated settings than vertical transmission. In future research, the impact of the feeding and drinking water systems as vehicles for the rapid spread of SI throughout the broiler house could be investigated. Finally, 94% of the SI isolates carried the megaplasmid, indicating a link between the presence of the megaplasmid and persistently contaminated farms.

## Figures and Tables

**Table 1:** Information on the farms: sizes of the sampled flocks at the five different farms (A–E); ages of the broiler houses at the moment of sampling (year); other animals on the farm; the hatcheries for the sampled rounds; who performed the C & D; duration of the vacancy; and if partial depopulation was applied.

Farm	Broiler House	Flock Size	Age (Years)	Other Animals	Hatchery	C & D	Duration Vacancy	Partial Depopulation
A	1	21,600	25	Goat, dog, cat	A	Farmer	2.5 weeks	No
	2	23,600	13		A	Farmer		
	3	31,300	2		B	External firm		
B	1	28,300	36	No	C	External firm	10 days	Yes
	2	28,300	30		C	External firm		
C	1	21,000	24	Dog	C	Farmer	1 week	Yes
	2	37,000	3		C	Farmer		
D	1	39,500	8	Dog	C	External firm	1 week	Yes
	2	39,500	8		C	External firm		
	3	75,000	5		C	External firm		
E	1	6125	31	No	C	Personnel	10–17 days	Yes
	2	6125	31		C	Personnel		
	3	6125	31		C	Personnel		
	4	6125	31		C	Personnel		

## Data Availability

The original contributions presented in this study are included in the article. Further inquiries can be directed to the corresponding author.

## Abbreviations

The following abbreviations are used in this manuscript:

BPW	Buffered peptone water
C & D	Cleaning and disinfection
MSRV	Modified semisolid Rappaport Vassiliadis
NCP	National control program
PFGE	Pulsed-field gel electrophoresis
SI	*Salmonella* Infantis
UPGMA	Unweighted-pair group method using arithmetic averages
XLD	Xylose lysine deoxycholate

## Appendix A

**Table A1:** Overview and details of the samples collected after C & D per broiler house. If two samples were taken (2×), this means that samples were taken in the anterior and posterior part of the broiler houses. In some cases, listed materials were not present and could not be sampled. Also, in some instances, extra samples of dirty materials were taken that are not listed here.

Broiler House		Anteroom		External Environment	
Surface type	Details	Surface type	Details	Surface type	Details
Floor (2×)	1 m^2^	Wheelbarrow	-	(Un)loading area	1 m^2^
Floor cracks (2×)	2 m	Brush	-	Mortality container inside	1 m^2^
Floor–wall connection (2×)	2 m	Bucket	-	Tractor shovel	-
Wall (2×)	1 m^2^	Shovel	-	Tractor wheels	-
Wall cracks (2×)	2 m	Squeegee	-	Mortality container outside	-
Ceiling (2×)	1 m^2^	Pressure washer	-		
Gate surface (2×)	1 m^2^	Floor	1 m^2^		
Hoppers (2×)	1 m^2^	Wall	1 m^2^		
Drink nipples + drip tray (2×)	3	Ceiling	1 m^2^		
Inside drinking line	10 cm	Access door anteroom	1 m^2^		
Outside downpipe feeding system (2×)	-	Access door broiler house	1 m^2^		
Inside downpipe feeding system (2×)	-	Boots	-		
Feed pans (2×)	3	Disinfection bath	0.5 m^2^		
Longitudinal fan	-	Sink and tap	0.5 m^2^		
Ridge fan (2×)	-	Control panel	1 m^2^		
Inlet valves (inside and outside) (2×)	3 (1 m^2^)				
Pipes on wall (2×)	2 m				
Pipes in stable (2×)	2 m				
Heating system (2×)	-				

**Table A2:** Overview and details of the samples collected during delivery of the one-day-old chickens.

Delivery Materials	
Surface type	Details
Truck wheels	4 wheels
Tail lift truck	1 m^2^
Cargo space truck	1 m^2^
Drive-in metal plate at gate	1 m^2^
Shoes of supplier	1 pair
Wheels of rack with crates	20 wheels
Inserts	20 samples
Crates	4 pooled samples

**Table A3:** Overview and details of the samples collected during rearing per broiler house. In some cases, listed materials were not present and could not be sampled. Also, in some instances, extra samples of dirty materials were taken that are not listed here.

Broiler House		Anteroom	
Surface type	Details	Surface type	Details
Wall + wall cracks + gate	1 m^2^ + 2 m + 1 m^2^	Wheelbarrow	-
Drink nipples + drip tray	4	Brush	-
Outside of feeding pans	4	Bucket	-
Inside of feeding pans	4	Shovel	-
Hoppers	2	Floor	1 m^2^
Inside and outside of downpipe feeding system	2	Wall	1 m^2^
Inlet valves (inside and outside)	3 (1 m^2^)	Access door anteroom	1 m^2^
Ridge fan	-	Access door broiler house	1 m^2^
Pipes on wall	2 m	Boots	-
Pipes in broiler house	2 m	Disinfection bath	0.5 m^2^
Heating system	-	Sink and tap	0.5 m^2^
Floor under plastic (2×)	1 m^2^	Control panel	1 m^2^
Overshoes	2 pairs		

**Table A4:** Overview of the *Salmonella*-contaminated samples taken at five broiler farms with a *Salmonella* Infantis contamination history during two production rounds. Also, dust samples, as overshoe samples (OS), were taken during production. During cleaning and disinfection (C & D) and delivery of the one-day-old chickens, only dust samples were taken. The locations that were sampled are the broiler houses (BHs), anterooms (ARs), and external environment (EE).

Farm	Location	C & D	Delivery	Week 1	Week 2	Week 3	Week 4	Week 5	Week 6
ROUND 1									
Farm A	BH 1	0/42	-	8/11 OS 2+	-	-	-	-	-
	BH 2	0/41	-	9/11 OS 2+	-	-	-	-	-
	BH 3	1/44	-	8/12 OS 2+	-	-	-	-	-
	AR 1	0/9	-	7/12	-	-	-	-	-
	AR 2	0/9	-	5/11	-	-	-	-	-
	AR 3	0/8	-	2/12	-	-	-	-	-
	EE	0/6	0/35	-	-	-	-	-	-
Farm B	BH 1	0/45	-	0/13	0/14	8/12 OS 2+	-	-	-
	BH 2	0/43	-	1/15	1/14	0/15	2/13 OS 2+	7/13 OS 2+	-
	AR 1	0/14	-	0/12	0/16	4/15	-	-	-
	AR 2	0/9	-	0/16	0/11	0/11	3/13	6/13	-
	EE	1/9	0/20	-	-	-	-	-	-
Farm C	BH 1	1/32	-	9/12 OS 2+	-	-	-	-	-
	BH 2	10/40	-	3/12 OS 2+	-	-	-	-	-
	AR 1	3/14	-	2/8	-	-	-	-	-
	AR 2	0/9	-	1/8	-	-	-	-	-
	EE	2/6	2/22	-	-	-	-	-	-
Farm D	BH 1	1/37	-	1/13 OS 1+	6/13 OS 2+	-	-	-	
	BH 2	0/39	-	0/13	2/13 OS 2+	-	-	-	-
	BH 3	0/38	-	0/15	0/18	-	0/13	0/16	-
	AR 1 and 2	1/13	-	3/13	2/15	-	-	-	-
	AR 3	0/12	-	0/11	0/8	-	1/10	0/9	-
	EE	0/19	0/33	-	-	-	-	-	-
Farm E	BH 1	0/19	-	0/13	1/12	1/13	0/13	0/13	0/13
	BH 2	1/20	-	0/13	0/12	0/13	0/13	0/13	0/13
	BH 3	0/20	-	0/13	0/12	0/12 OS 1+	0/13	0/13	0/13
	BH 4	0/18	-	0/13	0/12	0/13	0/13	0/13	0/13
	AR 1	0/10	-	0/7	0/9	0/10	0/11	0/12	0/13
	AR 2	0/9	-	0/8	0/10	1/13	0/14	0/12	0/13
	EE	1/7	0/18	-	-	-	-	-	-
ROUND 2									
Farm A	BH 1	0/37	-	8/13 OS 2+	-	-	-	-	-
	BH 2	1/41	-	4/13 OS 2+	-	-	-	-	-
	BH 3	1/37	-	0/14	0/14	0/14	0/12	1/10 OS 2+	-
	AR 1	0/10	-	4/12	-	-	-	-	-
	AR 2	0/8	-	1/11	-	-	-	-	-
	AR 3	0/9	-	0/10	0/10	0/10	0/10	0/10	-
	EE	0/6	0/21	-	-	-	-	-	-
Farm B	BH 1	2/47	-	5/13 OS 2+	-	-	-	-	-
	BH 2	5/52	-	3/15 OS 2+	-	-	-	-	-
	AR 1	2/13	-	3/14	-	-	-	-	-
	AR 2	0/9	-	0/4	-	-	-	-	-
	EE	1/9	1/19	-	-	-	-	-	-
Farm C	BH 1	0/31	-	0/11 OS 1+	8/10 OS 2+	-	-	-	-
	BH 2	0/30	-	0/12	0/12	0/12	-	0/12	-
	AR 1	0/10	-	0/8	3/8	-	-	-	-
	AR 2	0/8	-	0/9	0/9	0/9	-	0/9	-
	EE	1/5	1/19	-	-	-	-	-	-
Farm D	BH 1	0/43	-	0/13	0/14	0/14	2/13 OS 1+	-	-
	BH 2	0/28	-	0/13 OS 1+	0/14	0/14	11/12 OS 2+	-	-
	BH 3	0/37	-	0/15 OS 1+	2/12 OS 2+	-	-	-	-
	AR 1 and 2	0/12	-	0/14	0/12	1/11	2/5	-	-
	AR 3	1/12	-	0/9	0/9	-	-	-	-
	EE	1/15	0/29	-	-	-	-	-	-
Farm E	BH 1	0/17	-	-	0/12	-	-	0/12	-
	BH 2	0/17	-	-	0/12	-	-	0/12	-
	BH 3	0/18	-	-	0/12	-	-	0/12	-
	BH 4	0/17	-	-	0/12	-	-	0/12	-
	AR 1	0/11	-	-	0/9	-	-	0/9	-
	AR 2	0/10	-	-	0/10	-	-	0/10	-
	EE	0/3	-	-	-	-	-	-	-
